# Interaction between triglyceride-glucose index and thyroid hormones on coronary artery disease risk in patient with euthyroid

**DOI:** 10.3389/fendo.2023.1255656

**Published:** 2023-12-21

**Authors:** Li Li, Gaojun Cai, Wei Lu, Feng Li, Lei Yu, Jianqiang Xiao

**Affiliations:** Department of Cardiology, Wujin Hospital Affiliated to Jiangsu University, The Wujin Clinical College of XuZhou Medical University, Changzhou, Jiangsu, China

**Keywords:** free thyroxine, interaction, coronary artery disease, triglyceride glucose index, free triiodothyronine

## Abstract

**Background:**

Triglyceride-glucose (TyG) index is an effective indicator in indentifying in pre-diabetes, diabetes, and coronary artery disease (CAD). However, the value of TyG index combined with thyroid hormones (THs) to affect CAD has not been fully evaluated. Here, we investigated the association between TyG index and THs and further studied the impacts of TyG index and THs on CAD in euthyroid.

**Methods:**

Subjects (1,297) with euthyroid who underwent selective coronary angiography (CAG) were enrolled in the present study, including 893 patients with CAD and 404 controls. The association between TyG index and THs were analyzed by linear regression models. Multivariate logistic regression analysis was used to evaluate the interaction of TyG and THs with the risk of CAD. According to the cutoff value of free triiodothyronine (FT3), free thyroxine (FT4), thyroid-stimulating hormone (TSH), and TyG index, the patients were respectively separated into four groups: low TyG/FT3 (low or high), high TyG/FT3 (low or high), low TyG/FT4 (low or high), high TyG/FT4 (low or high), low TyG/TSH (low or high), high TyG/TSH (low or high).

**Results:**

The baseline analysis showed that FT4 level differs among the three groups according to the tertile of the TyG index. Multiple linear regression analysis revealed decreased serum FT3 level and serum FT4 level as an independent risk factor for elevated TyG index. After adjusting for confounding variables, multiple logistic regression analysis showed that patients with lower TyG index and higher FT3 level had an important protective effect on CAD when considering patients with lower TyG index and FT3 level as reference(OR = 0.536, 95% CI: 0.369–0.778, *P* = 0.001). Patients with higher TyG index and FT4 level (lower or higher) had a significantly increased risk of CAD (OR 1.656, 95% CI: 1.117–2.455; OR = 1.920, 95% CI: 1.279–2.848, respectively). The area under the curve for the combined diagnosis of CAD by TyG index and FT3 level is 0.615.

**Conclusions:**

These findings suggest that TyG is independently negatively correlated with FT3 or FT4 in euthyroid. In addition, there was a significant interaction between TyG index and THs on the risk of CAD.

## Background

Lipid and glucose disorders are common risk factors for coronary artery disease (CAD) (1). Insulin resistance (IR) is the major pathogenic factor of diabetes mellitus (DM) and plays an important role in the prognosis of CAD (2, 3). Triglyceride-glucose (TyG) index is an effective index, calculated as the product of triglyceride (TG) and fasting blood glucose (FBG), which has been found to be associated with IR, carotid artery plaque, arterial stiffness, and CAD (4–6). Observational studies have shown that a higher TyG index is associated with higher prevalence of cardiovascular disease in the general population. The prevalence of symptomatic CAD and the risk of major adverse cardiovascular and cerebrovascular events in patients with ST-segment elevation myocardial infarction (STEMI) are correlated with higher TyG index (7, 8). Eight cohort studies including 5,731,294 participants showed that higher TyG index was independently associated with an increased atherosclerotic cardiovascular disease risk. However, Alizargar et al. pointed out that TyG index as a valid index in patients with CAD is questionable, because it is affected by many factors including hyperlipidemia and diabetes.

Thyroid hormones (THs) have been linked with cardiac function, hepatic fatty acids, cholesterol synthesis, and metabolism. Low free triiodothyronine (FT3) level was negatively associated with the severity of CAD in euthyroid individuals (9). The TyG index, as an effective indicator of metablic syndrome, has been proven to be related to THs and THs sensitivity (10). Previous studies also investigated the relationship between THs and TyG index in non-diabetic adults and showed TyG index was negatively associated with FT4 level, positively associated with TSH level (11).

The interaction between TyG index and THs on the risk of CAD is not clear in euthyroid. Therefore, the aim of the present study is to examine the relationship between TyG index and CAD in euthyroid adults and investigated the interaction of TyG index and THs in promoting CAD.

## Methods

### Study design and subjects

**Figure 1:**
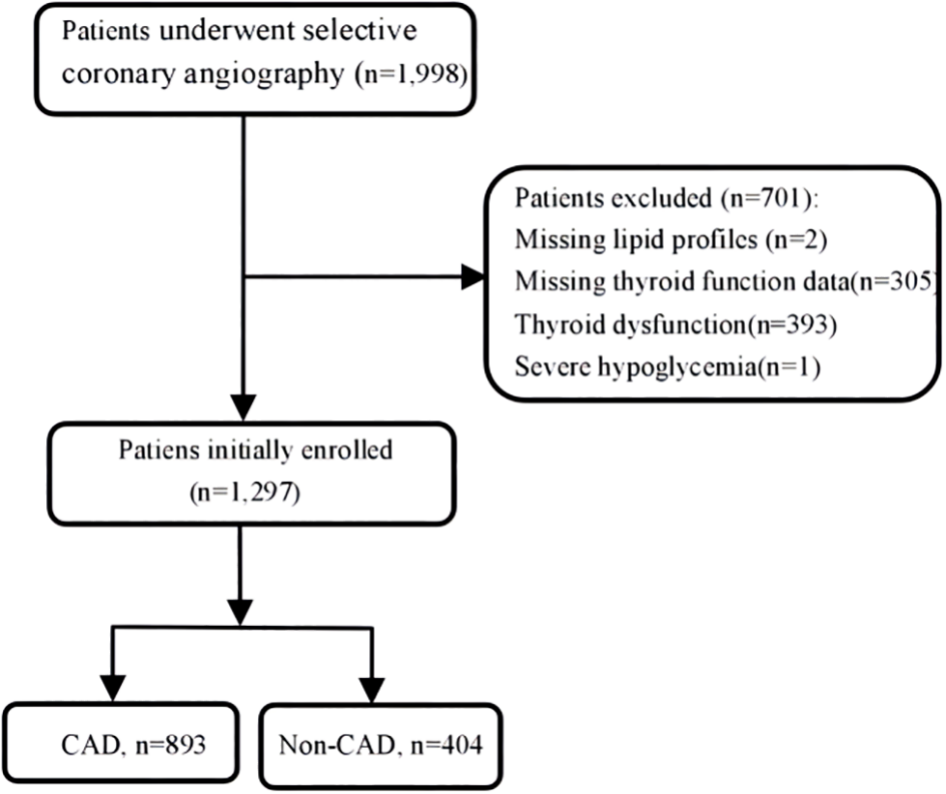
Flowchart of the study population enrollment.

There were overall 1,297 patients with euthyroid who underwent coronary angiography (CAG) at Wujin Hospital affiliated with Jiangsu University between June 2017 and March 2019 for angina pain and precordial discomfort on basis of positive noninvasive test results (i.e., electrocardiogram suggestive of ischemia, suspicious coronary computed tomography angiography, and so on). **Figure 1** shows the flowchart of the present study. The exclusion criteria were as follows: patients missing lipid profiles and thyroid function data, patients with thyroid dysfunction and receiving TH substitutions or antithyroid drugs, and patients with severe hypoglycemia. The study protocol was approved by the Ethics Committee of Wujin Hospital. This was a retrospective study, and informed consent could not be obtained from every participant, which was approved by the Ethics Committee of Changzhou Wujin Hospital (no. 201787).

### Data collection

At least 8h fasting venous blood samples were collected on the second day of admission. The basic clinical information, namely, age, sex, height, weight, medication record, history of DM, and hypertension were collected. Blood biochemical measurements, namely, serum creatinine (Scr), FBG, total cholesterol (TC), TG, low-density lipoprotein cholesterol (LDL-C), high-density lipoprotein cholesterol (HDL-C), apolipoprotein A-I (apo A-I), and apolipoprotein B (apo B) were measured by an automated analyzer (AU5800 Beckman Coulter, Beckman Coulter Inc., Brea,California, USA), and thyroid function was carried out by a fully automatic immunoassay analyzer (Dxl 800 Beckman Coulter, Beckman Coulter Inc., USA).

The TyG index was calculated as the Ln [fasting TGs (mg/dl) × fasting glucose (mg/dl)]/2.

### Diagnostic criteria

CAD was defined in accordance with the 1979 WHO diagnostic criteria (12). CAD was defined as at least one major coronary vessel stennosis exceeding 50% (left main, left anterior descending, left circumflex, right coronary artery, and large branches). All patients underwent a CAG examination after admission. The CAG examinations were performed using the Judkin technique via the radial or femoral artery. Angiograms were analyzed by at least two experienced doctors who were blinded to this study. Non-CAD was defined as those without coronary disease and the presence of coronary stenosis less than 50%, regardless of symptom (13).

The reference intervals of normal thyroid function were as \follows: FT3, 3.5 pmol/L to 6.5 pmol/L; FT4, 11.5 pmol/L to 22.7 pmol/L; thyroid-stimulating hormone (TSH), 0.55 mIU/L to 4.78 mIU/L. In this study, euthyroidism was defined as having no history of hyperthyroidism or hypothyroidism and having normal levels of FT3, FT4, and TSH.

### Statistical analysis

Statistical Package for the Social Sciences software version 20.0 (SPSS Inc. Chicago, IL, USA) and GraphPad Prism 8 software were used to perform statistical analysis. Categorical variables were compared using the chi-square test and expressed as frequencies and percentages. Continuous variables were tested for normality with Kolmogorov–Smirnov statistics. Skew distribution variables are presented as the medians (interquartile ranges), and differences in variables among groups were compared using the Kruskal–Wallis H test. The baseline data of subjects were grouped according to the tertile of TyG index in this study. In accordance with the optimal cutoff values of TyG (8.78), FT3 (4.62 pmol/L), FT4 (16.85 pmol/L), and TSH(1.87mIU/L), participants were divided into four groups, respectively: TyG−/FT3−(Q1);TyG−/FT3+(Q2); TyG+/FT3−(Q3);TyG+/FT3+(Q4); TyG−/FT4−(Q1);TyG−/FT4+(Q2);TyG+/FT4−(Q3);TyG+/FT4+(Q4); and TyG−/TSH−(Q1);TyG−/TSH+(Q2);TyG+/TSH− (Q3);TyG+/TSH+(Q4). Logistic regression analyses were performed to estimate the interaction between TyG index and THs on the risk of CAD, and confounding factors, namely, age, sex, and body mass index (BMI) were adjusted. Odds ratio (OR) and the corresponding 95% confidence interval (CI) were calculated after controlling for confounding variables. *P* < 0.05 was considered significant.

## Results

### The characteristics of the study subjects according to TyG index

The baseline characteristics of the study population are displayed in **Table 1**. The current study finally enrolled 1,297 subjects who were stratified into three groups according to the tertile level of TyG index (T1:TyG index < 8.54; T2:8.54 ≤ TyG indx ≤ 9.06; T3:TyG indx > 9.06). BMI, FPG, HbAIc, TC, TG, LDL-C, apo A-I, apo B and TyG of the subjects gradually increased with the increase of TyG index, while age, Scr, HDL-C, and FT4 gradually decreased with the increase of TyG index. In addition, the higher proportion of DM, hypertension, and CAD was found in the higher TyG index group. However, there is no difference in FT3- and TSH-level among the three groups.

**Table 1:** Baseline and biochemical characteristics in patients.

	Total(n=1297)	TyG index			P-value
		T1(n=432)	T2(n=430)	T3(n=435)	
Age, years	64.18(53.9-74.4)	66.37(56.7-75.9)	64.47(54.86-74.1)	61.7(50.9-72.5)	<0.001
Male, n (%)	881(67.9%)	307(71%)	285(66.2%)	289(66.4%)	0.225
Take lipid-lowering drugs,n(%)	240(18.5%)	99(22.9%)	74(17.2%)	67(15.4%)	0.172
BMI(kg/m^2^)	24.79(21.53-28.05)	23.65(20.64-26.66)	24.91(21.71-28.11)	25.81(22.61-29.01)	<0.001
Scr(mmol/L)	71.74(53.65-89.83)	73.16(55.57-91.20)	71.67(53.65-89.69)	70.38(52.04-88.72)	0.078
FBG(mmol/L)	6.07(3.99-8.16)	5.10(4.18-6.02)	5.69(4.33-7.05)	7.41(4.69-10.13)	<0.001
HbA1c(mmol/L)	6.25(4.86-7.64)	5.72(4.89-6.55)	6.05(4.94-7.16)	6.97(5.23-8.71)	<0.001
TG(mmol/L)	4.35(2.86-5.84)	3.89(2.92-4.86)	4.39(2.39-6.40)	4.76(3.59-5.86)	<0.001
TC(mmol/L)	1.89(0.43-3.35)	0.98(0.75-1.21)	1.57(1.23-1.91)	3.10(1.05-4.97)	<0.001
HDLC(mmol/L)	1.09(0.83-1.36)	1.18(0.90-1.46)	1.09(0.85-1.33)	1.00(0.78-1.23)	<0.001
LDL-C(mmol/L)	2.71(1.78-3.63)	2.40(1.58-3.22)	2.78(1.86-3.70)	2.94(2.01-3.87)	<0.001
ApoA(mmol/L)	1.23(0.99-1.47)	1.21(1.07-1.37)	1.23(1.01-1.45)	1.24(0.98-1.50)	0.192
ApoB (mmol/L)	0.87(0.59-1.16)	0.72(0.51-0.94)	0.86(0.63-1.10)	1.04(0.73-1.36)	<0.001
TyG index	8.86(8.20-9.52)	8.20(7.96-8.44)	8.78(8.64-8.92)	9.60(9.10-10.10)	<0.001
FT3(pmol/L)	4.64(4.05-5.23)	4.65(4.03-5.26)	4.67(4.09-5.25)	4.61(4.04-5.18)	0.651
FT4(pmol/L)	16.99(14.67-19.33)	17.23(14.87-19.60)	16.98(14.77-19.19)	16.76(14.39-19.13)	0.012
TSH (mIU/L)	1.88(1.28-2.74)	1.77(1.25-2.60)	1.90(1.25-2.77)	1.93(1.31-2.85)	0.265
fT3/fT4	0.27(0.23-0.32)	0.27(0.23-0.32)	0.27(0.23-0.33)	0.28(0.24-0.33)	0.124
Diabetes mellitus, n (%)	328(25.5%)	47(10.8%)	96(22.3%)	185(42.5%)	<0.001
Hypertension,n (%)	897(69.1%)	271(62.7%)	302(70.2%)	324(74.5%)	<0.001
CAD, n (%)	893(68.0%)	278(64.3%)	296(68.8%)	319(73.3%)	0.007

BMI Body mass index, Scr serum creatinine, FBG fasting blood. BMI, Body mass index; Scr, serum creatinine; FBG, fasting blood glucose; HbA1c, glycated haemoglobin; TG, triglyceride; TC, total cholesterol; HDL-C, high-density lipid cholesterol; LDL-C, low-density lipid cholesterol; apoA-I, apolipoprotein A-I; apoB, apolipoprotein B; TyG, the triglycerideglucose index; FT3, free triiodothyronine; FT4, free thyroxine; TSH, thyroid stimulating hormone; CAD, coronary artery disease.

### The association between TyG and THs in euthyroid adults

In multivariate linear regression model, the TyG index was used as the dependent variable, and age, BMI, uric acid, FBG, and THs parameters were used as independent variables. The results showed that FT3 and FT4 levels were negatively associated with the TyG index (*P* < 0.05) in **Table 2**. When FT3 and FT4 levels increased by 1 pmol/L, the TyG index reduced by 0.056 and 0.078, respectively. BMI, uric acid, and FBG were positively correlated with the TyG index. The TyG index increased by 0.543 for every 1 mmol/L increase in the FBG level.

**Table 2:** Multiple linear regression analysis for the association between TyG index and FT3 or FT4 level.

Variable	Standardized coefficients (β)	T	*P*
FT3	-0.056	-2.436	0.015
FT4	-0.078	-3.432	<0.001
TSH	0.025	1.147	0.251
Age	-0.171	-7.712	<0.001
BMI	0.159	7.057	<0.001
Uric acid	0.127	5.822	<0.001
FBG	0.543	24.70	<0.001

FT3, Free Triiodothyronine; FT4, Free Thyroxine; TSH, thyroid-stimulating hormone; BMI, Body mass index; FBG, fasting blood glucose.

### Logistic regression analysis of interaction between TyG index and THs on the risk of CAD

In order to explore the interaction of TyG index and THs on the risk of CAD, odds ratio (OR) and *P*-values are presented in **Table 3**. Taking TyG−/FT3− group as a reference, the risk of CAD in TyG−/FT3+ group decreased significantly by 46.4% after adjusting for age, sex, and BMI. Similarly, patients with higher levels of TyG, regardless of their FT4 and TSH levels, indicate a higher risk of CAD (OR = 1.656 for lower FT4; OR = 1.920 for higher FT4, OR = 1.762 for lower TSH; OR = 1.692 for higher TSH). As shown in **Table 4**, the stratified analysis observed patients with TyG−/FT4+ aged ≤60 years old have a lower risk of CAD, and this result was not shown in those aged >60 years old. The risk of CAD in TyG+/FT4+ group with BMI ≤24 kg/m^2^ (OR = 3.230, 95% CI = 1.503–6.938) is higher than those in TyG+/FT4− group with BMI >24 kg/m^2^ in **Table 5**.

**Table 3:** Interaction of the TyG and THs with the risk of CAD using binary logistic regression.

Interaction	No.	UnadjustedOR(95%CI)	*P*	*Adjusted OR(95%CI)	*P*
TyG/FT3					
Q1 Q2 Q3 Q4	329 326 331 311	Reference 0.543(0.378-0.780) 1.270(0.849-1.902) 1.005(0.709-1.570)	0.001 0.245 0.790	Reference 0.536(0.369-0.778) 1.447(0.953-2.196) 1.138(0.743-1.745)	0.001 0.083 0.552
TyG/FT4					
Q1	317	Reference		Reference	
Q2	334	0.955(0.669-1.364)	0.799	0.957(0.667-1.373)	0.811
Q3	333	1.460(0.999-2.133)	0.051	1.656(1.117-2.455)	0.012
Q4	313	1.681(1.131-2.500)	0.010	1.920(1.279-2.848)	0.002
TyG/TSH					
Q1	316	Reference		Reference	
Q2	329	1.053(0.754-1.471)	0.764	1.115(0.794-1.566)	0.528
Q3	338	1.677(1.173-2.397)	0.005	1.762(1.220-2.545)	0.003
Q4	313	1.479(1.050-2.085)	0.025	1.692(1.182-2.422)	0.004

TyG, Triglyceride glucose; FT3, Free Triiodothyronine; FT4, Free Thyroxine; TSH, thyroid-stimulating hormone.

*Adjusted for age, sex, BMI.

**Table 4:** Interaction of the TyG and THs with the risk of CAD according to age.

	TyG/FT3				TyG/FT4				TyG/TSH			
	Age≤ 60years		Age>60years		Age≤ 60years		Age>60years		Age≤ 60years		Age>60years	
Inter-action	OR*(95%CI)	P	OR*(95%CI)	P	OR*(95%CI)	P	OR*(95%CI)	P	OR*(95%CI)	P	OR*(95%CI)	P
Q1	Reference		Reference		Reference		Reference		Reference		Reference	
Q2	0.459 (0231-0.913)	0.027	0.597 (0.379-0.942)	0.026	0.461 (0.241-0.881)	0.019	1.359 (0.866-2.132)	0.182	0.998 (0.535-1.862)	0.995	1.142 (0.755-1.726)	0.529
Q3	2.601 (1.155-5.859)	0.021	1.083 (0.666-1.762)	0.747	1.736 (0.879-3.430)	0.112	1.480 (0.910-2.405)	0.114	3.928 (2.012-7.666)	0.000	1.155 (0.741-1.801)	0.525
Q4	1.325 (0.644-2.724)	0.445	1.025 (0.597-1.762)	0.927	2.177 (1.090-4.351)	0.028	1.662 (1.006-2.748)	0.048	2.617 (1.414-4.844)	0.002	1.318 (0.843-2.060)	0.227

TyG, Triglyceride glucose index; FT3, Free Triiodothyronine; FT4, Free Thyroxine; TSH, thyroid-stimulating hormone.

*Adjusted for age, sex.

**Table 5:** Interaction of the TyG and THs with the risk of CAD according to BMI.

	TyG/FT3				TyG/FT4				TyG/TSH			
	BMI≤ 24kg/m^2^		BMI> 24kg/m^2^		BMI≤ 24kg/m^2^		BMI> 24kg/m^2^		BMI≤ 24kg/m^2^		BMI> 24kg/m^2^	
Inter-action	OR*(95%CI)	*P*	OR*(95%CI)	*P*	OR*(95%CI)	*P*	OR*(95%CI)	*P*	OR*(95%CI)	*P*	OR*(95%CI)	*P*
Q1	Reference		Reference		Reference		Reference		Reference		Reference	
Q2	0.514 (0.308-0.859)	0.011	0.540 (0.311-0.938)	0.029	0.705 (0.427-1.165)	0.173	1.368 (0.805-2.327)	0.247	1.201 (0.751-1.921)	0.444	1.035 (0.633-1.693)	0.891
Q3	2.513 (1.233-5.124)	0.011	1.071 (0.616-1.862)	0.808	1.719 (0.874-3.378)	0.116	1.691 (1.035-2.762)	0.036	1.818 (1.019-3.244)	0.043	1.751(1.082-2.829)	0.023
Q4	1.568 (0.749-3.284)	0.233	0.949 (0.545-1.651)	0.853	3.230 (1.503-6.938)	0.003	1.620 (0.977-2.689)	0.062	1.976 (1.096-3.563)	0.023	1.602 (1.006-2.551)	0.047

TyG, Triglyceride glucose index; FT3, Free Triiodothyronine; FT4, Free Thyroxine; TSH, thyroid-stimulating hormone.

*Adjusted for age, sex.

### The predictive efficacy of combination of TyG and FT3 or FT4

**Figure 2:**
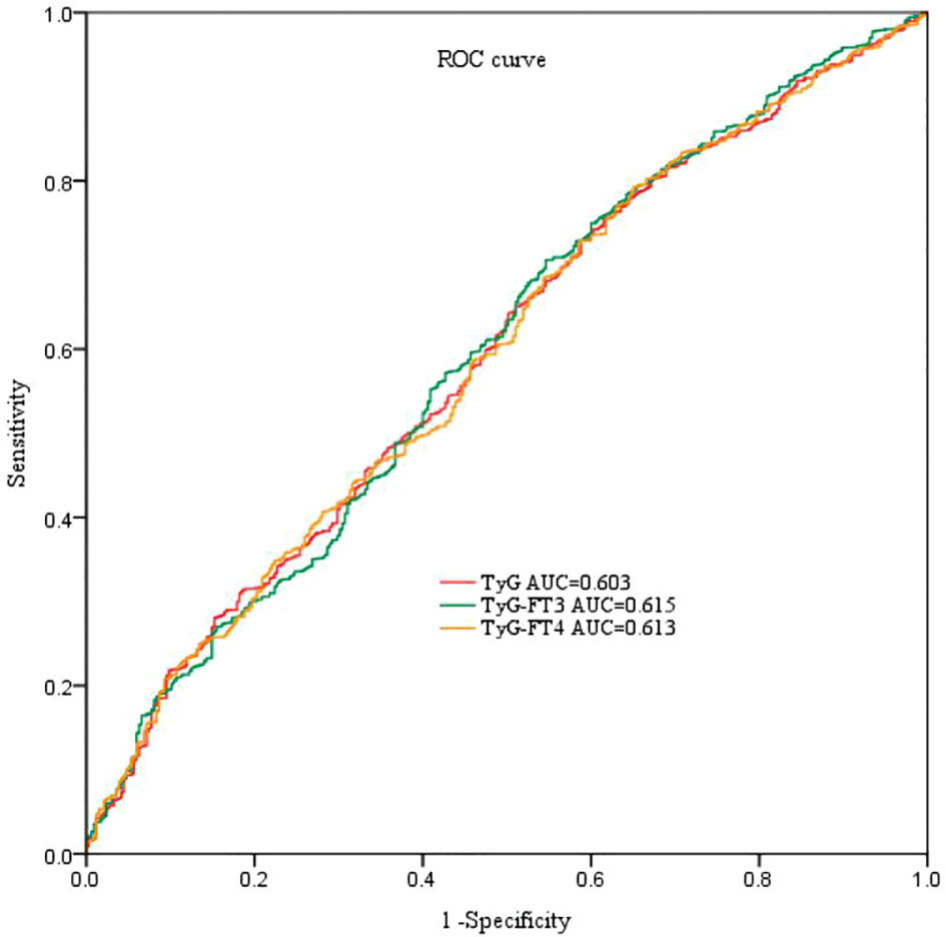
ROC curve analyses for predicting CAD. ROC, receiver-operating characteristic; AUC, area under the curve; TyG index, triglyceride-glucose index; FT3, free triiodothyronine; FT4, free thyroxine.

Receiver-operating characteristic curve was performed to evaluate the predictive efficacy of combination of TyG and FT3 or FT4 in **Figure 2**. The AUC of TyG to predict CAD was 0.603. The AUC of TyG combined with FT3 and FT4 are 0.615 and 0.613, respectively. Compared with TyG alone, adding FT3 to TyG resulted in an increase in AUC and an improvement in the predictive ability of the new model, but the difference was not statistically significant (NRI = 2.24%, *P* = 0.32).

## Discussion

There is relatively little research on the relationship between TyG and THs in euthyroid. The present study firstly demonstrated that TyG index was negatively associated with FT3 or FT4 in euthyroid subjects. We also found an interaction between TyG and THs, which has a significant impact on CAD. Meanwhile, we explored the predictive value of combined indicators based on TyG index and FT3 or FT4 for CAD.

CAD is the main cause of death worldwide (14). IR and metabolic syndrome (including hypertension, hyperlipidemia, hyperuricemia, and obesity) are associated with DM and atherosclerosis (15–17). A research from Thailand demonstrated hypertriglyceridemia is an independent risk factors for future DM and positively correlated with incidence of DM during a 10-year follow-up period (18). A large prospective cohort study also showed that fasting glucose within normal range, following certain trajectories, may increase risk of developing CAD (19). Researchers paid more attention to TyG index in recent years, which is the product of fasting plasma glucose and TG levels (7). Many studies reported that TyG index is a valuable marker for metabolic disorders (20, 21). Currently, there are several studies on TyG index and cardiovascular disease, but the results are inconsistent. Luo E et al. pointed TyG index is positively related to the incidence of major adverse cardiovascular and cerebrovascular events in STEMI patients (8). Zhao et al. showed that TyG index was significantly correlated to arterial stiffness, cardiometablic risk factors, chronic kidney disease, microalbuminuria, and macrovascular damage (22). Meanwhile, recent evidence also suggested that TyG index can increase the prevalence of in-stent restenosis in patient with acute coronary syndrome after drug stent implantation. The number of coronary vessels and SYNTAX score increased with the elevated of TyG index, making it possible for TyG index to serve as an indicator for predicting the severity of CAD and cardiovascular outcomes in patients with non-ST-segment elevation acute coronary syndrome (23, 24). However, an article published in 2020 analyzed that TyG index may not the best index in predicting CAD and may be affected by confounding factors such as blood glucose and dyslipidemia (25).

THs play an important role in maintaining myocardial remodeling, IR, glucose and lipid metabolism (26, 27). The research on THs and dyslipidemia, diabetes and obesity is gradually increasing. Patients with CAD often have thyroid dysfunction. Previous studies have found that low FT3 levels were negatively correlated with CAD and major adverse cardiovascular events (28). The TyG index has been studied as an alternative indicator of IR in many fields. It was firstly reported in non-diabetic euthyroid adults from Korea Survey. The results showed a significant negative correlation between TyG index and FT4 level in the male population. Meanwhile, this study also indicated a negative correlation between TyG and homoeostatic model assessment of insulin resistance (HOMA-IR). In another study, an inverse correlation was found between FT4 and HOMA-IR (29). The possible reason is that THs cannot only directly affect IR but also indirectly affect IR through BMI, body composition, gender, and so forth. After adjusting for confounding factors, our study found that TyG index was inversely associated with FT3 or FT4 level in euthyroid, respectively.

Higher FT3 level is a protective factor for CAD and correlated with decreased cardiovascular mortality and all-cause mortality, even in individuals with normal thyroid function (11). FT3 was found to be positively correlated with total and LDL cholesterol concentrations in a study of 1,372 individuals with normal thyroid function. This may be strong evidence to support our results. In our study, participants with lower levels of TyG index and higher levels of FT3 had a lower risk of CAD whether before or after adjusting for confounding factors. In age stratified analysis, the higher risk of CAD was observed in individuals under 60 years of old with higher TyG index and lower FT3 level, but this phenomenon has not been found in the elderly. Previous studies have reported that lower FT3 with normal range does not increase the presence and severity of CAD in old patients. There may be related to the lifestyle changes of young people nowadays and a downregulated in sensitivity to THs in older subjects.

The higher risk of CAD was observed in populations with higher levels of TyG and lower levels of FT4, and this risk is more significant in populations with both higher TyG and FT4. From the further analysis, we also found that the increased incidence of CAD was observed in patients with BMI ≤24 kg/m^2^. Interestingly, this result was not obtained when BMI >24kg/m^2^. These observations strongly suggest that body weight may interfere with the interaction between TyG index and FT4 on CAD. The thyroid and adipose tissue play important roles in the metabolism of the body. The fat mass directly increases the activity of deiodinase, resulting in an increase in the peripheral conversion rate of T4 in T3 (30). The activity of deiodinase also decreases with age, leading to a decrease in the conversion rate from T4 to T3 (31). It seems reasonable to change the body and thyroid status through appropriate exercise and dietary structure. This observation suggests that understanding the age/BMI-related THs status in patients with euthyroid is necessary. It may guide us in better managing cardiovascular risk or controlling CAD risk in patients with thyroid diseases undergoing medication. More studies are necessary to confirm these findings and underlying mechanism.

TyG has been studied as a predictive indicator in multiple fields, and adding the TyG index to the baseline risk model can significantly improve prediction accuracy. In this study, we firstly explored the predictive ability of TyG combined with thyroid function in CAD, but the results are not optimistic.

However, several limitations should be acknowledged. First, as a single-center, retrospective and observational study, the sample size of the study is small. Second, although CAD-related risk factors were adjusted for, we still cannot exclude the possibility of residual or unrecorded confounding factor such as life style.

## Conclusion

These findings suggest that TyG is independently negatively correlated with FT3 or FT4 in euthyroid. In addition, there was a significant interaction between TyG index and THs on the risk of CAD. Age and body mass index have a certain role in TyG and TH regulation of CAD, which provides clues for clinical treatment of coronary heart disease.

## Data availability statement

The original contributions presented in the study are included in the article/supplementary material. Further inquiries can be directed to the corresponding author.

## Ethics statement

The studies involving humans were approved by the Ethics Committee of Changzhou Wujin Hospital (no.201787). The studies were conducted in accordance with the local legislation and institutional requirements. The human samples used in this study were acquired from primarily isolated as part of your previous study for which ethical approval was obtained. Written informed consent for participation was not required from the participants or the participants’ legal guardians/next of kin in accordance with the national legislation and institutional requirements.

## Author contributions

LL: Writing – original draft, Conceptualization. GC: Writing – review & editing. WL: Investigation, Writing – review & editing. FL: Investigation, Writing – review & editing. LY: Data curation, Formal analysis, Writing – review & editing. JX: Data curation, Formal analysis, Writing – review & editing.
